# Evaluation of retinal vascularization in retinopathy of prematurity regressed after intravitreal ranibizumab monotherapy or without treatment based on fluorescein angiography

**DOI:** 10.1038/s41598-023-47212-7

**Published:** 2023-11-15

**Authors:** Lei Wu, Manhong Li, Liang Wang, Hongxiang Yan, Ziyi Zhou, Jing Fan, Yi Zhou, Kaili Gou, Changmei Guo, Yusheng Wang, Zifeng Zhang

**Affiliations:** Department of Ophthalmology, Eye Institute of Chinese PLA, Xijing Hospital, Fourth Military Medical University, Xi’an, 710032 Shaanxi Province China

**Keywords:** Retinopathy of prematurity, Retinal diseases, Retinopathy of prematurity

## Abstract

To investigate the fluorescein angiography (FA) findings and compare the extent of retinal vascularization in retinopathy of prematurity (ROP), recovered after intravitreal ranibizumab (IVR) monotherapy and those regressed spontaneously. Infants with a history of ROP who underwent FA between April 2018 and November 2021 were retrospectively included. The patients were divided into two groups based on whether they had received IVR (IVR group) or had ROP that regressed spontaneously without treatment (untreated group). The differences between the two groups in zone II ROP were also compared, to equalize the subgroups as much as possible in terms of disease severity. FA findings were recorded. The extent of vascularization was measured by the ratio of the distance from the center of the disk to the border of the vascularized zone (DB) and the distance from the center of the disk to the center of the fovea (DF). The width of the persistent avascular retina (PAR) was counted by disc diameters (DD). One hundred and ten eyes of 55 infants were included in the IVR group and 76 eyes of 38 babies in the untreated group. The ratio of abnormal shape of vessels was significantly higher in the IVR group than in the untreated group (50.9% vs. 35.5%; *P* = 0.038), while the linear choroidal filling pattern, tortuosity of vessels over the posterior pole, dye leakage, anomalous branching of vessels, circumferential vessels, arteriovenous shunt, abnormal capillary bed, and macular abnormalities were similarly. There was a smaller temporal DB/DF ratio (4.48 vs. 4.63; *P* = 0.003) and greater PAR (2.63 vs. 1.76; *P* < 0.001) in the IVR group compared to the untreated group. In zone II ROP, the progression of retinal vascularization was significantly larger in the IVR group than that in the untreated group (*P* = 0.003), while no statistical differences were observed in FA features, the DB/DF ratio, and PAR between the two subgroups. The residual vascular abnormalities and PAR may be common results of ROP regression. The DB/DF ratio of 4.0 temporally and 3.3 nasally could be used as the preliminary indicators for safe retinal vascularization in the completion of ROP regression.

## Introduction

Retinopathy of prematurity (ROP) is a retinal vascular proliferative disease in low gestational age (GA) and birth weight (BW) infants. It is the leading cause of preventable childhood blindness worldwide^1^. Most ROP cases tend to regress spontaneously, while severe cases require timely treatment to prevent retinal detachment and blindness^2^. To date, laser photocoagulation is still the standard procedure for ROP treatment. It has also been proved that intravitreal injection of anti-vascular endothelial growth factor (VEGF) agents was effective for ROP^3^. In recent years, anti-VEGF agents, such as ranibizumab, conbercept, and bevacizumab, are widely used in the treatment of ROP^4–6^.

Similar to spontaneous regression of ROP, an important feature of ROP treated with anti-VEGF agents is that the retinal vessels initiated at the ridge could continuously develop to the peripheral retina and complete retinal vascularization^7^. However, it was shown that some fundus abnormalities persisted in patients with ROP even after the completion of regression, such as abnormal branching, arteriovenous (AV) shunt, capillary dropout, and incomplete retinal vascularization, etc^8,9^. Although it is unknown whether anti-VEGF agents suppress retinal vascularization or not, the prevalence of these vascular abnormalities is due to anti-VEGF therapy or the regression of ROP itself, which remains to be controversial, and the long-term implications are yet not known^10^. Further research is needed to evaluate the significance of the abnormal vascular lesions and the necessity for prophylactic treatment of abnormalities such as dye leakage and persistent avascular retina (PAR). Fluorescein angiography (FA) has been proven to be an effective tool that could accurately assess vascular abnormalities and the extent of retinal vascularization in ROP^11^. In the present study, we retrospectively compared the fundus and FA images between type 1 ROP^12^ recovered following intravitreal injection of ranibizumab (IVR) monotherapy without reactivation and ROP regressed spontaneously without treatment. The aim is to precisely evaluate retinal vascularization in ROP regression with FA and to provide a reference for guiding treatment paradigms and monitoring protocols.

## Methods

### Study design and patients

This was a single-center retrospective study performed in the department of Ophthalmology, Xijing Hospital, Fourth Military Medical University. It was approved by the Ethics Committee of Xijing hospital (KY20202099-C-1) and followed the principles of the Helsinki Declaration. Informed consent was obtained from all parents. Infants with a history of ROP who underwent FA between April 2018 and November 2021 were included. The patients with ROP were divided into two groups based on whether they had received IVR (IVR group) or regressed spontaneously without treatment (untreated group). Patients with any of the following conditions were excluded: (1) patients with ROP reactivation; (2) patients who received laser treatment; (3) patients who were not followed up until the completion of regression. In our hospital, a thorough fundus examination was performed by two senior professional pediatric ophthalmologists using RetCam 3 (Clarity Medical Systems, Pleasanton, California, USA). The infants who were diagnosed as ROP with RetCam3 must be verified by another senior professional pediatric ophthalmologist using indirect ophthalmoscope (Keeler Company, Britain). Fundus images of each infant were recorded in RetCam 3. ROP was classified according to the International Classification of Retinopathy of Prematurity, Third Edition^2^. Type 1 ROP was treated with IVR at a concentration of 0.25 mg/0.025 ml, and then followed up every 1–4 weeks or every 1–3 months according to fundus appearance. Infants who did not meet treatment criteria were followed up every 2–4 weeks or every 1–3 months till the completion of regression or longer. To evaluate the development of retinal vessels, FA was performed with RetCam 3 after 60 weeks postmenstrual age (PMA) when retinal vascularization terminated after treatment or spontaneous regression.

### Data collection

**Figure 1:**
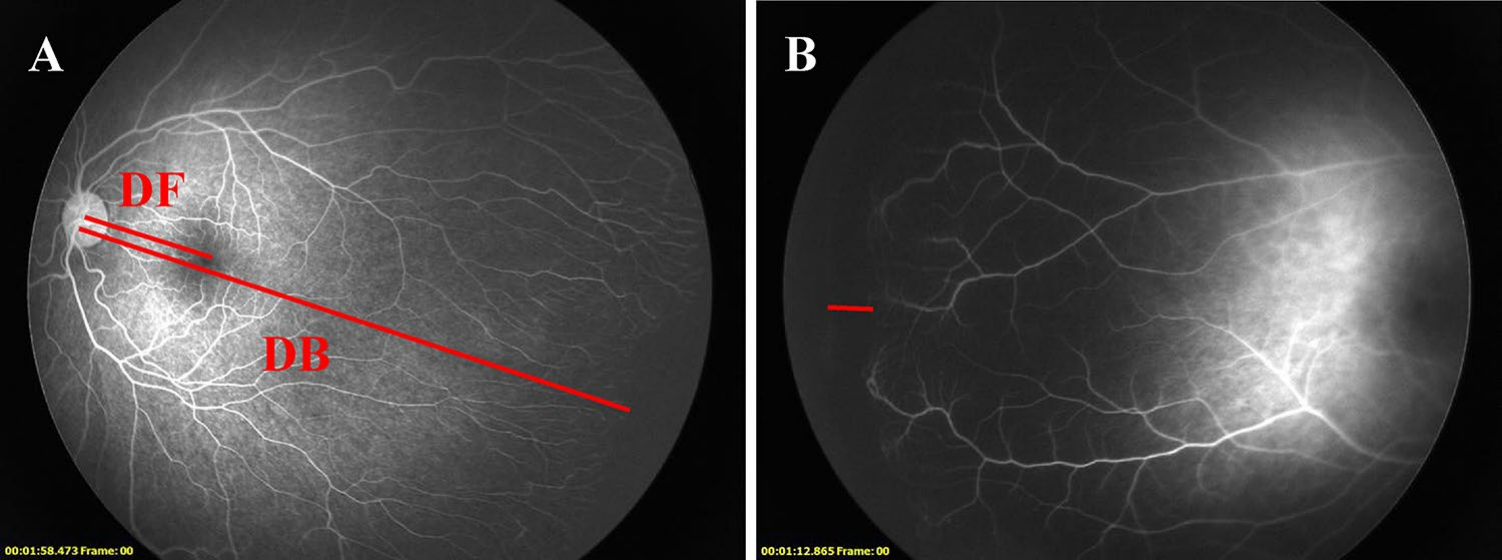
Measurement of the distance from the center of the disk to the border of the vascularized zone (DB) and the distance from the center of the disk to the center of the fovea (DF) (**A**). Measurement of the width of persistent avascular retina (the red line) (**B**).

Baseline characteristics of the enrolled patients including GA, BW, sex, fetus number (single or multiple), and delivery type (spontaneous vaginal delivery or cesarean section) were recorded. Date of the first ROP screening, IVR treatment, each follow-up, and FA performing were recorded. Fundus characteristics of ROP at the time of IVR were recorded, including zone, stage, and the presence of plus disease. FA was performed with 10% sodium fluorescein (0.1 mL/kg, IV), followed by an isotonic saline flush and observed for at least 5 min. FA findings were documented, including choroidal filling pattern, tortuosity of arteries over the posterior pole, leakage, anomalous branching of vessels, abnormal shape of vessels, circumferential vessels, AV shunt, abnormal capillary bed, and macular abnormalities. During the FA process, the choroidal filling pattern is usually observed clearly in the eye examined firstly, and the morphology of the choroidal blood vessels of the other eye examined later would be blurred by the background fluorescence. The first eye examined in each patient was selected for the analysis of the choroidal filling pattern, to reduce the errors in FA interpretation. The extent of retinal vascularization was determined by the ratio of the distance from the center of the disk to the border of the vascularized zone (DB) and the distance from the center of the disk to the center of the fovea (DF) (Fig. 1A)^13^. The width of the PAR in the temporal retina was recorded by disc diameters (DD) (Fig. 1B). In addition, patients with clear fundus images and complete data were included to calculate the progression of retinal vascularization after IVR and spontaneous regression. The initial temporal vascular location (from the center of the optic disc to the initial demarcation line or ridge through the fovea) was noted by DD on the fundus images before treatment or the onset of spontaneous regression, and the final vascular termini were recorded in the same way on FAs. The distance between the two sites was the progression of retinal vascularization. All measurements were performed with Image J 1.8.0 software to minimize error. To delve further into the retinal vascularization in ROP regression, we compared the features in FA findings and the completion of retinal vascularization between the IVR group and the untreated group. The differences between the two groups in zone II ROP were also compared, to equalize the subgroups as much as possible in terms of disease severity.

### Statistical analysis

All statistical analyses were performed with SPSS statistical software (version 26.0). The categorical variables were presented as counts and percentages. The continuous variables all conformed to a normal distribution, expressed as mean ± standard deviation (SD). The chi-square test or *Fisher*’s exact probability test was used for categorical variables. Continuous variables were analyzed with the Mann–Whitney *U*-test or Student's *t*-test. A two-tailed *P* value < 0.05 was recognized as statistically significant.

## Results

A total of 196 eyes of 98 patients, who were treated with IVR, underwent FA examinations from April 2018 to November 2021, of which 86 eyes of 43 infants (43.9%) with reactivated ROP were excluded, since most of them were retreated with photocoagulation. Then the other 110 eyes of 55 patients were included in the IVR group. The reason for the high constituent ratio of reactivated ROP is that many reactivated infants have been referred to our center for FA examination and retreatment. Seventy-six eyes of 38 patients were included in the untreated group. All the patients are free of reactivation until the final follow-up. Baseline characteristics of the infants are listed in Table 1. The GA of the IVR group and the untreated group was 28.69 weeks and 30.88 weeks respectively, and the difference was statistically significant (*P* < 0.001). The BW of the IVR group and the untreated group was 1179.64 g and 1494.74 g respectively, with statistically significant difference (*P* < 0.001). In terms of fetus number, the untreated group (81.6%) consisted of a higher proportion of singletons than the IVR group (58.2%) (*P* = 0.018). There were no statistically significant differences in sex and mode of delivery between the two groups (*P* = 0.576, *P* = 0.870). FA was performed at PMA of 73.59 weeks in the IVR group and 69.75 weeks in the untreated group, and there was no statistical difference between them (*P* = 0.116). The PMA at final follow-up in the IVR group was 119.00 weeks, which was significantly later than 94.60 weeks in the untreated group (*P* < 0.001).

**Table 1:** Baseline characteristics of ROP infants treated with intravitreal ranibizumab monotherapy and spontaneous regression.

Baseline characteristics	IVR group (n = 55)	Untreated group (n = 38)	*P* value
GA, weeks	28.69 ± 1.91	30.88 ± 2.51	< 0.001
BW, g	1179.64 ± 322.29	1494.74 ± 428.45	< 0.001
Fetus number, n(%)			
Single	32 (58.2)	31 (81.6)	0.018
Multiple	23 (41.8)	7 (18.4)	
Delivery pattern, n(%)			
SVD	28 (50.9)	20 (52.6)	0.870
Cesarean section	27 (49.1)	18 (47.4)	
Sex, n(%)			
Male	35 (63.6)	22 (57.9)	0.576
Female	20 (36.4)	16 (42.1)	
PMA at treatment, weeks	39.51 ± 3.00		
PMA at FA, weeks	73.59 ± 12.46	69.75 ± 9.81	0.116
PMA at final follow-up, weeks	119.0 ± 27.61	94.60 ± 23.24	< 0.001

*BW* birth weight; *GA* gestational age; *SVD* spontaneous vaginal delivery; *PMA* postmenstrual age.

Baseline diagnoses are summarized in Table 2. In the IVR group, ROP stage 2 + or 3 + in zone II were diagnosed in 41 infants, and ROP stage 2 + or 3 + in zone I in 14 infants. In the untreated group, 21 infants were diagnosed as ROP stage 2 or 3 in zone II without plus disease, and 17 infants were diagnosed as ROP stage 2 in zone III without plus disease.

**Table 2:** Baseline diagnoses of ROP infants treated with intravitreal ranibizumab monotherapy and spontaneous regression.

Baseline diagnoses	IVR group (n = 55)	Untreated group (n = 38)
Zone I stage 2 + /stage 3 +	14	0
Zone II stage 2 + /stage 3 +	41	0
Zone II stage 2/stage 3	0	21
Zone III stage 2/stage 3	0	17

**Figure 2:**
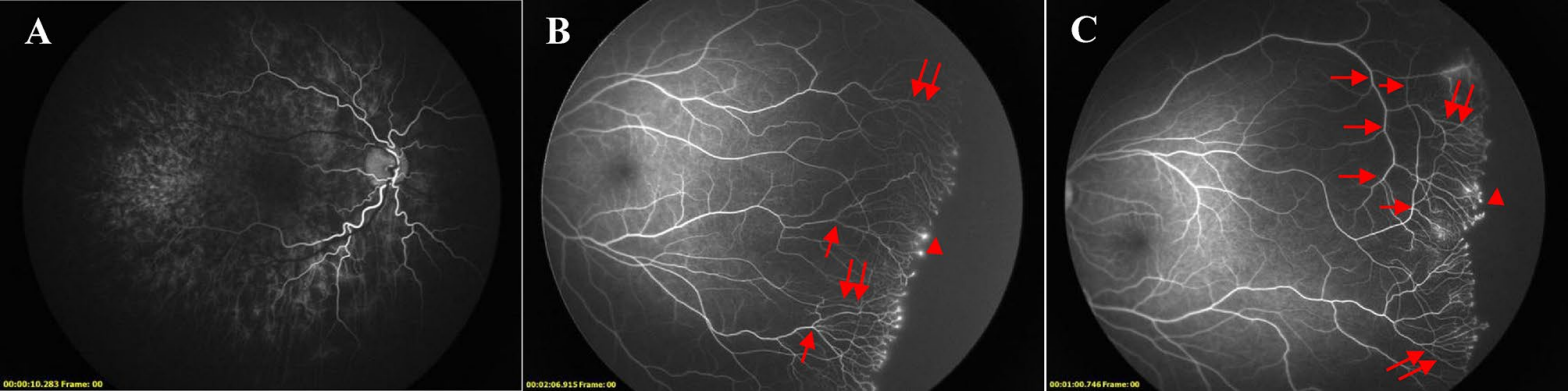
Former 30 week-old 1710 g male infant treated with intravitreal ranibizumab at 40 weeks for Type 1 ROP. Fluorescein angiography at 85 weeks of postmenstrual age demonstrating the linear choroidal filling pattern and tortuosity of arteries over the posterior pole (**A**). Former 28 week-old 800 g female infant from the spontaneous regression group. Fluorescein angiography at 68 weeks of postmenstrual age demonstrating leakage (closed arrowhead), anomalous branching of vessels (arrow), and abnormal shape of (double arrow) vessels detected in areas from initial ridge to vascular termini (**B**). Former 28 week-old 1100 g female infant treated with intravitreal ranibizumab at 40 weeks for Type 1 ROP. Fluorescein angiography at 78 weeks of postmenstrual age demonstrating punctate hyperfluorescent lesions (arrowhead), abnormal shape of vessels (double arrow), and circumferential vessels (arrow) (**C**).

**Figure 3:**
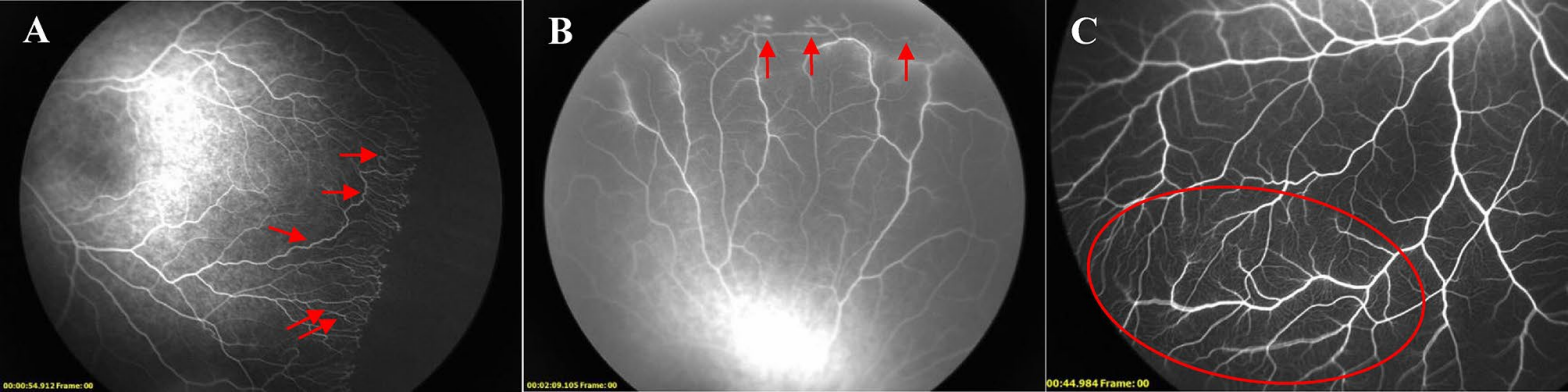
Former 28 week-old 1340 g male infant treated with intravitreal ranibizumab at 36 weeks for Type 1 ROP. Fluorescein angiography at 61 weeks of postmenstrual age demonstrating circumferential vessels (arrow) and abnormal shape of vessels (double arrow) detected in areas from initial ridge to vascular termini (**A**). Former 28 week-old 1100 g male infant from the spontaneous regression group. Fluorescein angiography at 70 weeks of postmenstrual age demonstrating arteriovenous shunt (arrow) (**B**). Former 31 week-old 1300 g male infant treated with intravitreal ranibizumab at 39 weeks for Type 1 ROP. Fluorescein angiography at 68 weeks of postmenstrual age demonstrating abnormal lacy capillary bed (seen best in the circular regions) (**C**).

**Figure 4:**
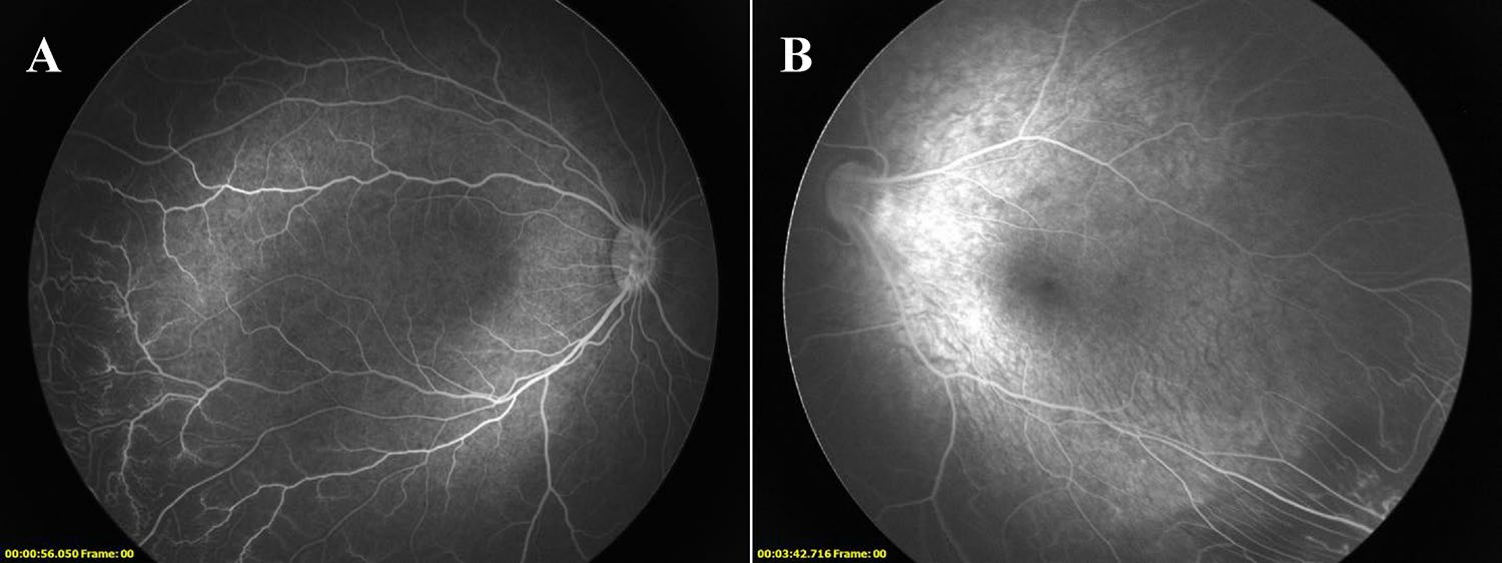
Former 29 week-old 980 g male infant from the spontaneous regression group. Fluorescein angiography at 63 weeks of postmenstrual age demonstrating the absence of foveal avascular zone and hypoperfusion (**A**). Former 30 week-old 1100 g male infant from the spontaneous regression group. Fluorescein angiography at 66 weeks of postmenstrual age demonstrating macular dragging (**B**).

Fluorescein angiography findings of retinal vascularization in infants with ROP are summarized in Table 3. The linear choroidal filling pattern was noted in 10 eyes (18.2%) in the IVR group and 7 eyes (18.4%) in the untreated group (Fig. 2A). In the posterior retina, tortuosity of arteries was detected in 33 eyes (30.0%) in the IVR group, compared with 28 eyes (36.8%) in the untreated group (Fig. 2A). In the peripheral retina, FA findings observed in both groups included punctate or linear dye leakage during the late phase of FA at the vascular-avascular junction, anomalous branching of vessels, abnormal shape of vessels, circumferential vessels, AV shunt, and abnormal capillary bed. Dye leakage was present on FA in 32 eyes (29.1%) and 23 eyes (30.3%) in the IVR and untreated groups (Fig. 2B,C). The anomalous branching of vessels showed the disappearance of the normal cone-shaped bifurcations, replaced by three or even four bifurcations, which was present in 72 eyes (65.5%) and 43 eyes (56.6%) of the two groups (Fig. 2B). The abnormal shape of vessels showed the disappearance of normal physiological bending of peripheral blood vessels and presents a straight shape (Figs. 2B,C and 3A). We noted the abnormal shape of vessels in 56 eyes (50.9%) and 27 eyes (35.5%) in the IVR and untreated groups. The thick circumferential vessels often originated from the initial ridge and grew to vascular termini (Figs. 2C and 3A). AV shunt were mostly located in the superior or inferior quadrants along with the terminal position of retinal vascularization (Fig. 3B). In the IVR group, we observed circumferential vessels in 64 eyes (58.2%) and AV shunt in 27 eyes (24.5%). In the untreated group, circumferential vessels were detected in 40 eyes (52.6%) and AV shunt in 16 eyes (21.1%). The abnormal capillary bed was characterized by loss or lacy of capillary bed, and shown in 56 eyes (50.9%) and 41 eyes (53.9%) of the two groups (Fig. 3C). The macular abnormalities showed the absence of foveal avascular zone and/or hypoperfusion, which were present in 43 eyes (39.1%) and 28 eyes (36.8%) in the IVR and untreated group (Fig. 4A). One patient failure to follow-up on time developed macular dragging in the left eye (Fig. 4B), was followed up for 12 months without any other treatment, and fundus examination revealed that the lesion did not progress. There were no significant differences in FA findings except for the abnormal shape of vessels (*P* = 0.038). Furthermore, there was no significant differences in FA features, when compared the results of FA findings in ROP infants of zone II in the IVR group and the untreated group (all *P* > 0.05).

**Table 3:** Fluorescein angiography findings of ROP patients treated with intravitreal ranibizumab monotherapy and spontaneous regression.

Fluorescein angiography findings, n(%)	IVR group			Untreated group			*P*_1_ value	*P*_2_ value
	Total	Zone I	Zone II	Total	Zone II	Zone III		
	(n = 110)	(n = 28)	(n = 82)	(n = 76)	(n = 42)	(n = 34)		
Linear choroidal filling pattern^a^	10 (18.2)	2 (14.3)	8 (19.5)	7 (18.4)	3 (14.3)	4 (23.5)	0.977	0.735^b^
Tortuosity of vessels over the posterior pole	33 (30.0)	6 (21.4)	27 (32.9)	28 (36.8)	18 (42.9)	10 (29.4)	0.329	0.276
Leakage	32 (29.1)	8 (28.6)	24 (29.3)	23 (30.3)	19 (45.2)	4 (11.8)	0.863	0.077
Anomalous branching of vessels	72 (65.5)	23 (82.1)	49 (59.8)	43 (56.6)	31 (73.8)	12 (35.3)	0.221	0.122
Abnormal shape of vessels	56 (50.9)	22 (78.6)	34 (41.5)	27 (35.5)	18 (42.9)	9 (26.5)	0.038	0.882
Circumferential vessels	64 (58.2)	13 (46.4)	51 (62.2)	40 (52.6)	26 (61.9)	14 (41.2)	0.454	0.975
Arteriovenous (AV) shunt	27 (24.5)	8 (28.6)	19 (23.2)	16 (21.1)	8 (19.0)	8 (23.5)	0.579	0.599
Abnormal capillary bed	56 (50.9)	15 (53.6)	41 (50.0)	41 (53.9)	27 (64.3)	14 (41.2)	0.683	0.130
Macular abnormalities	43 (39.1)	14 (50.0)	29 (35.4)	28 (36.8)	17 (40.5)	11 (32.4)	0.756	0.577

Linear choroidal filling pattern^a^ : The first eye of each patient to examine was included for the analysis.

^b^*Fisher’s* exact probability tests was used.

P_1_ value: IVR group versus Untreated group.

P_2_ value: Zone II ROP in the IVR group versus zone II ROP in the untreated group.

Measurement of retinal vascularization is summarized in Table 4. The DB/DF ratio and the residual PAR were measured in all the patients, and the progression of retinal vascularization was measured in 80 eyes of 40 patients in the IVR group and 28 eyes of 14 patients in the untreated group, of which 62 eyes of 31 patients and 18 eyes of 9 patients were identified as ROP in zone II. In the IVR group, the DB/DF ratio was 4.48 in the temporal retina, and 3.73 in the nasal, while in the untreated group, the ratio was 4.63 in the temporal retina and 3.78 in the nasal. There was a statistical difference in the temporal side between the two groups (*P* = 0.003), but there was no statistical difference in the nasal retina (*P* = 0.172). The residual PAR in the temporal retina was 2.63 DD and 1.76 DD in the IVR group and the untreated group, respectively, and the difference was statistically significant (*P* < 0.001). The proportions of patients with the width of PAR > 2.0 DD were higher in the IVR group than in the untreated group (*P* = 0.001). The progression of retinal vascularization in the temporal retina was 2.59 DD in the IVR group and 1.23 DD in the untreated group, with statistically significant difference (*P* < 0.001). The subgroup analysis showed that there were no significant differences in the DB/DF ratio and width of PAR between the two subgroups (all *P* > 0.05). However, in zone II ROP, the progression of retinal vascularization was significantly larger in the IVR group than that in the untreated group (*P* = 0.003).

**Table 4:** Measurement of retinal vascularization of ROP patients treated with intravitreal ranibizumab monotherapy and spontaneous regression.

		DB/DF ratio , mean ± SD		The width of PAR (DD, mean ± SD)	The distance from vascular termini to the initial ridge (DD, mean ± SD)	The width of PAR (n, % )	
		The temporal side	The nasal side			> 2.0 DD	≤ 2.0 DD
IVR group	Total	4.48 ± 0.36	3.73 ± 0.22	2.63 ± 1.16	2.59 ± 1.23	63 (57.3)	47 (42.7)
	Zone I	4.39 ± 0.32	3.71 ± 0.26	3.20 ± 1.05	3.54 ± 1.19	25 (89.3)	3 (10.7)
	Zone II	4.52 ± 0.37	3.74 ± 0.21	2.44 ± 1.13	2.32 ± 1.11	38 (46.3)	44 (53.7)
Untreated group	Total	4.63 ± 0.29	3.78 ± 0.23	1.76 ± 1.00	1.23 ± 1.10	25 (32.9)	51 (67.1)
	Zone II	4.58 ± 0.35	3.76 ± 0.23	2.16 ± 1.01	1.38 ± 1.32	20 (47.6)	22 (52.4)
	Zone III	4.70 ± 0.18	3.81 ± 0.23	1.23 ± 0.69	0.98 ± 0.50	5 (14.7)	29 (85.3)
*P*_*1*_ value		0.003	0.172	< 0.001	< 0.001	0.001	
*P*_*2*_ value		0.389	0.694	0.238	0.003	0.893	

DB = the distance from the center of the optic disc to the border of the vascularized zone; DF = the distance from the center of the disc to the fovea.

*DD* disc diameter, *PAR* persistent avascular retina.

*P*_1_ value: IVR group versus Untreated group.

*P*_2_ value: Zone II in the IVR group versus zone II in the untreated group.

## Discussion

Although anti-VEGF agents are widely used in the treatment of ROP, problems regarding the abnormalities during pathological regression and the criteria of retinal vascularization remain to be discussed^13^. Similarly, these issues also exist in ROP spontaneously regressed without treatment^9^. The application of FA in pediatric patients allows us to observe the abnormalities of retinal blood vessels, and to quantify the retinal vascularization more clearly. To our knowledge, this is the largest case series to systematically evaluate the retinal vascularization in ROP regressed after IVR or without treatment based on FA features.

The dilation and tortuosity of the posterior retinal vessels were named plus disease, which is one of the indications of severe ROP^2^. In the two groups, the tortuous arteries without vasodilation in the posterior retina were detected in some of the patients. Similar to the study of Mansukhani et al., this feature was equally common in both groups^9^. Retinal blood vessel tortuosity and dilation could be the result of increased VEGF expression^14^. It was indicated that these eyes still have a higher VEGF burden even though ROP regressed. Mild pre-plus or no plus disease existed in the untreated group before the onset of regression, while plus disease was present before treatment in the IVR group. The accumulation of VEGF in some eyes may have continued after the onset of spontaneous regression or after treatment-induced regression. It was concluded that the presence of tortuosity in the posterior retina following intravitreal bevacizumab (IVB) associated with elevated VEGF level was an early predictor of potential reactivation^15^. Caution and clinical judgment are required to determine when follow up examination can be safely terminated in cases with tortuosity.

Leakage can be detected in patients after anti-VEGF treatment or spontaneous regression^16^. In this study, slight late leakage was seen in 32 eyes (29.1%) in the IVR group, compared to 23 eyes (30.3%) in the untreated group, which is consistent with the previous study^9^. It suggested that the presence of slight dye leakage after completion of regression is one of the common fundus changes. The incidence of leakage was similar between the two groups, leakage frequently occurred in zone II ROP of the untreated group rather than that of the IVR group, although differences were not statistically significant. It was indicated that ranibizumab decreased the angiogenic activity and had a positive effect on reducing the occurrence of dye leakage by binding VEGFA.

It was shown in our findings that vascular abnormalities, including anomalous branching, abnormal shape of vessels, circumferential vessels, AV shunt, and abnormal capillary bed were common in zone II ROP between the two groups. It suggested that anti-VEGF treatment neither reduced nor increased the occurrence of vascular abnormalities, and we agree with the authors that peripheral vascular abnormalities probably occur as a result of ROP itself rather than anti-VEGF treatment^9,17^. More abnormal shape of vessels was noted in the IVR group. The difference may be due to the fact that these manifestations were very common in zone I ROP of the IVR group, but less in zone III of the untreated group. Interestingly, the vascular abnormalities were often detected in areas from the initial ridge to vascular termini after retinal vascularization ceased. It was therefore speculated that the vascular abnormalities originated from acute ROP, and outwarded toward the retinal periphery with the onset of regression and retinal vascularization. The results regarding vascular abnormalities were different from the findings of the previous study. Vural et al.^18^ reported that vascular anomalies in ROP with anti-VEGF treatment were significantly less than those with spontaneous regression, and speculated that the reason might be a possible positive effect of anti-VEGF agents to reduce vascular anomalies. It may be related to the composition of the patients and the different analyzing methods. In addition, the PMA at FA was relatively early in their study, long-term progression in the vascularization process of the retina in some cases may also have contribution^19^.

PAR and DB/DF ratios are a pair of indicators that validate each other, which further enhanced the credibility of our findings. Our results showed that the PAR greater, the extent of the temporal retinal vascularization smaller, despite a greater progression of retinal vascularization in the IVR group than those in the untreated group. It was supposed that the difference may be due to the inconsistent severity of the disease between the two groups. The comparison between the two groups in zone II ROP showed that the extent of retinal vascularization and PAR were similar in the IVR group and the untreated group. Therefore, we agreed with previous authors that anti-VEGF treatment was not to cause cessation in vascular progression^20^. While, we speculated that the severity of ROP, especially the location of the initial ridge before the onset of regression, is a more significant factor than IVR treatment itself on the extent of retinal vascularization.

In recent years, complications such as retinal detachment and retinal tears following anti-VEGF treatment or regressed spontaneously have prompted clinicians to prefer prophylactic peripheral laser for patients with PAR greater than 2.0 DD, a sign of incomplete retinal vascularization, and with fluorescein leakage after 60 weeks PMA^21–23^. Wang^24^ and associates found that late peripheral leakage was seen in 32.53% of the eyes in normal adults. In fact, part of leakage may be due to endothelial cell dysfunction when new vessels formed during vascular development, and not all lesions with leakage during FA require treatment in clinical practice. Moreover, numerous studies reported that part of eyes treated with anti-VEGF agents failed to achieve completed vascularization^25–29^. In our cohort, PAR greater than 2.0 DD was detected in 63 (57.3%) eyes in the IVR group and 25 (32.9%) eyes in the untreated group. Celiker and associates concluded that PAR may not be a major risk factor alone, and not all patients with incomplete retinal vascularization need prophylactic laser application^30^. Both in theory and on the basis of available data, late reactivation is possible in ROP treated with anti-VEGF agents^31,32^, and late-onset complications may occur in ROP regressed spontaneously^3,22^. To minimize the impact or poor prognosis, long-term follow-up was carried out in our clinic work. In the present study, the results from follow-up confirmed that no reactivation and severe complications occurred in the eyes with leakage or PAR greater than 2.0 DD. We suggested that neither leakage nor PAR greater than 2.0 DD is a reliable indicator of late reactivation and retreatment.

Since it is difficult to identify the ora serrata on FA images, Lorenz et al.^13^ developed the method of DB/DF ratio based on the distances of morphological structures and considered eyes with a vascularized zone of more than the DB/DF ratio 4 temporally and 3 nasally to be fully vascularized. The statistical analysis had not been performed due to the small sample size in their study. In the present study, the unilateral 95% medical reference value method defined as the mean—1.64SD was employed to quantify the retinal vascularization. Based on this method, the DB/DF ratio less than 3.89 in the temporal retina and 3.37 in the nasal could be considered abnormal after IVR. Accordingly, the DB/DF ratio less than 4.15 in the temporal retina and 3.40 in the nasal should be considered abnormal after the completion of spontaneous regression. The purpose of applying Image J software was to minimize error in measurement. However, the 3.89 and 3.37 DB/DF ratio is not clinically distinguishable from 4.15 and 3.40 using indirect ophthalmoscope or RetCam. Aside from statistical reasoning, we suggest that the ratio of 4.0 temporally and 3.3 nasally should be a practical threshold value of completed vascularization by combining the definition of zones in ROP, and the presented DB/DF ratios may also be used as the preliminary indicators. Only 10% of eyes (n = 11) in the IVR group and 2.6% of eyes (n = 2) in the untreated group included in this study would be classified as incompletely vascularized by this reference value. It suggests that these patients may require careful and periodic follow-up, while the follow-up frequency of remaining patients could be appropriately reduced or terminated. No infants developed reactivation, which further confirms the reference value should provide an acceptable safety margin.

Several limitations exist in the study. First, this is a single-center retrospective study, and only patients who underwent FA during follow-up were included, which may lead to selection bias. Further prospective studies are needed to verify. Second, although the follow-up period in our study is relatively long, there is a lack of follow-up for older or adult cases. We therefore cannot draw conclusions on long-term outcome and safety. In addition, infants treated with IVR (the more severe ROP in general with high risks of reactivation after anti-VEGF treatment) required longer follow-up time than those with spontaneously regressed ROP, so the PMA at final follow-up of the IVR group was approximately 24.4 weeks (5 months) older than those in the control group. Finally, some reactivated ROP after ranibizumab were excluded. The IVR group cannot fully represent all the patients following IVR therapy.

In conclusion, this is a large series of FA-based systematic investigation of the fundus appearance and extent of retinal vascularization of ROP regressed after anti-VEGF therapy or without treatment. Furthermore, our study introduces the reference value for incomplete retinal vascularization. These results provide us with a better understanding of the regression patterns of ROP and may help to avoid unnecessary fundus examinations and treatments. Further studies are needed to assess long-term outcomes associated with vascular abnormalities and PAR. Prospective multicenter FA studies would provide precise guidance for follow-up schedules and preventive treatment protocols to inhibit reactivation.

## Data Availability

The datasets used or analyzed during the current study are available from the corresponding author on reasonable request.
